# Asymmetric hybridization in *Cordulegaster* (Odonata: Cordulegastridae): Secondary postglacial contact and the possible role of mechanical constraints

**DOI:** 10.1002/ece3.4368

**Published:** 2018-09-14

**Authors:** Emanuela Solano, Sönke Hardersen, Paolo Audisio, Valentina Amorosi, Gabriele Senczuk, Gloria Antonini

**Affiliations:** ^1^ Department of Biology and Biotechnology ‘‘Charles Darwin’’ University of Rome “La Sapienza” Rome Italy; ^2^ Centro Nazionale per lo Studio e la Conservazione della Biodiversità Forestale “Bosco Fontana” Carabinieri Marmirolo Mantua Italy

**Keywords:** asymmetric introgression, *Cordulegaster boltonii*, *Cordulegaster trinacriae*, geometric morphometrics, hybrid zone, phylogeography, species boundaries

## Abstract

Two *Cordulegaster* dragonflies present in Italy, the Palaearctic and northern distributed *Cordulegaster boltonii* and the endemic to the south of the peninsula *Cordulegaster trinacriae*, meet in central Italy and give rise to individuals of intermediate morphology. By means of mitochondrial and nuclear markers and of Geometric Morphometrics applied to sexual appendages, we defined i) the geographical boundaries between the two species in Italy and ii) we determined the presence, the extent, and the genetic characteristics of the hybridization. Genetic data evidenced asymmetric hybridization with the males of *C. trinacriae* able to mate both interspecifically and intraspecifically. The results contrast with expectations under neutral gene introgression and sexual selection. This data, along with the morphological evidence of significant differences in size and shape of sexual appendages between the males of the two species, seem indicative of the role of mechanical constraints in intraspecific matings. The origin of the two species is dated about to 1.32 Mya and the hybridization resulted related to range expansion of the two species after Last Glacial Maximum and this led to the secondary contact between the two taxa in central Italy. At last, our results indicate that the range of *C. trinacriae*, a threatened and protected species, has been moving northward probably driven by climate changes. As a result, the latter species is currently intruding into the range of *C. boltonii*. The hybrid area is quite extended and the hybrids seem well adapted to the environment. From a conservation point of view, even if *C. trinacriae* has a strong genetic identity, the discovery of hybridization between the two species should be considered in a future species management.

## 1 INTRODUCTION

The evolutionary history of European biota is closely linked to postglacial expansion, which has structured the current genetic distribution of species (Hewitt, 2000; Schmitt, 2007). The Last Glacial Maximum (LGM), the latest event of global cooling, shaped the phylogeny and phylogeography of many extant populations, and the effects of the postglacial processes have been investigated thoroughly. The first reviews by Hewitt (1999, 2000, 2001) highlighted that most postglacial colonization events in animals and plants followed a similar pattern of expansion from refugia.

A common phenomenon in the model of expansion after the LGM contraction of the Northern Hemisphere is the formation of hybrid zones after secondary contact between populations (Hewitt, 2001; Schmitt, 2007). Although it is often difficult to determine whether secondary contact occurred in an area, most cases reported in the literature proved to be secondary contacts related to climatic fluctuations in the Quaternary (37%, reviewed by Barton & Hewitt, 1985; but see also Taberlet, Fumagalli, Wust‐Saucy, & Cosson, 1998; Davis & Shaw, 2001; Currat, Ruedi, Petit, & Excoffier, 2008). The comparison between expansion patterns has revealed the presence in Europe of hybridization “hotspots” for thermophilous taxa (called suture zones, Remington, 1968; Hewitt, 2000 for a review; Stewart & Lister, 2001) and one of these is located in northern Italy (Schmitt, 2007; Taberlet et al., 1998). The Alps were a barrier for species dispersal (Schmitt, 2007; Slechtová, Bohlen, Freyhof, Persat, & Delmastro, 2004) and the south of Italy has been indicated as a glacial refugium for several taxa (Canestrelli, Sacco, & Nascetti, 2012; Schmitt, 2007; Taberlet et al., 1998). It is interesting that the existence of refugia in sub‐Alpine xerothermic areas has also been reported (Bhagwat & Willis, 2008; Kaltenrieder et al., 2009).

When two allopatric populations come into secondary contact and the reproductive barriers fail, the formation of hybrid zones takes place, which can be more or less stable and extended (Hayashi, Dobata, & Futahashi, 2005; Sánchez‐Guillén et al., 2014; Wolf, Takebayashi, & Rieseberg, 2001). Interspecific hybridization is recognized as one of the potential drivers of speciation (Abbott et al., 2013; Barton, 2001; Jiggins & Mallet, 2000; Mallet, 2007). In fact, introgression, that is, acquisition of new genes through hybridization and backcrossing (Andersson, 1949), could be an important source of new variability and subsequent evolution. Hybridization can be symmetric (bidirectional), if males and females of both species are involved in heterospecific mating, or asymmetric (unidirectional), if one sex is predominantly involved in heterospecific mating. This is reflected in differential transfer of genes (hybrids contain a discordant mitochondrial/nuclear pattern) and of specific matrilinear and patrilinear markers.

When a species colonizes a new area where a sister species is already present, a massive introgression of neutral genes between the intrusive and the local species may take place. A number of factors can promote unidirectional hybridization (Canestrelli et al., 2016; Currat et al., 2008; Grant & Grant, 1997; Wirtz, 1999) and these are different in case of selective/not selective processes. If interbreeding is not prevented or if selective processes are not present (including asymmetric mating preference), introgression occurs almost exclusively from the local to the intrusive species, irrespective of the relative densities of the two species (Currat et al., 2008). In the presence of selective processes, mechanical and ethological barriers are among the main prezygotic causes of asymmetric hybridization. Mechanical incompatibility of structures involved in mating is an efficient prezygotic isolation mechanism during tandem formation and copulation. This is an important constraint to interspecific reproduction in Odonata (Monetti, Sánchez‐Guillén, & Cordero Rivera, 2002; Paulson, 1974; Sánchez‐Guillén, Wellenreuther, Cordero‐Rivera, & Hansson, 2011). Under sexual selection, when females of one species are locally rare and cannot find suitable (i.e., conspecific) males, they eventually mate with males of the locally common species (Turgeon, Stoks, Thum, Brown, & McPeek, 2005). Thus, if an immigrant species invades an area of a closely related species, the rare females mate with both abundant and rare males to enhance their fitness (Wirtz, 1999). These circumstances are in contrast to what occurs in the absence of selective processes (Currat et al., 2008). At last, Canestrelli et al. (2016) highlighted that, during range expansion, asymmetric hybridization can be influenced by sexual preference, as a character linked to dispersal propensity, and spatial sorting of traits could affect the direction and extent of gene exchange. In some cases, the tendency to mate assortatively could increase at the expansion front and differential introgression could be favoured (examples in Canestrelli et al., 2016).

From a morphological point of view, in extant hybrid zones relying on gene flow, individuals may show admixture of morphological characters or a spatial transition between hybridizing forms of the two parental species. In other cases, character displacement evolves as an adaptive response of one or both species to the hybridization and when the speciation has occurred the hybrid zone is expected to disappear over time (Barton & Hewitt, 1985).

The genus *Cordulegaster* (Odonata: Cordulegastridae) has an Holarctic distribution, comprises 29 species and four of these are present in Italy: *C*. *heros* Theischinger, 1979, *C*. *bidentata* Sélys 1843 and the two sister species *C*. *boltonii* (Donovan, 1807) and *C*. *trinacriae* Waterston, 1976. *Cordulegaster boltonii*, a species with Palaearctic distribution, is present in north‐central Italy while *C. trinacriae* is endemic to Sicily and to the southern Italian regions Basilicata, Calabria, Campania, and Molise (Riservato, Fabbri, et al., 2014). The two species can easily be distinguished by morphological features (Boudot, 2001; Dijkstra & Lewington, 2006). Froufe, Ferreira, Boudot, Alves, & Harris (2013) confirmed the validity of *C. trinacriae* as a distinct species but investigated only a limited number of populations from Italy, and they did not include samples from the central part of the peninsula where the two sister species are likely to be in contact. Riservato, Fabbri, et al., 2014 presumed that *C*. *boltonii* and *C. trinacriae* can be sympatric in central Italy, and both species have been caught in Gerano (Rome) (Galletti & Pavesi, 1985). However, no detailed data are available from the contact area.

In the case of natural hybridization, it is important to consider if one of the two species involved is endangered and to establish the possible consequences for conservation (Genovart, 2009). Interspecific hybridization in Odonata is common and well documented, that is, in *Coenagrion* (Lowe, Harvey, Thompson, & Watts, 2008), *Mnais* (Hayashi, Dobata, & Futahashi, 2004; Hayashi et al., 2005), *Calopteryx* (Tynkkynen et al., 2008) and *Ischnura* (Leong & Hafernik, 1992; Monetti et al., 2002; Sánchez‐Guillén et al., 2011). In the genus *Cordulegaster*, one putative hybrid between *C. bilineata* and the sister species *C*. *diastatops* has recently been identified with molecular tools (Pilgrim, Roush, & Krane, 2002). Therefore, it would be important to investigate the contact zone between *C. boltonii* and *C. trinacriae*, as the latter species has been listed as “near threatened” in the Italian and the European IUCN Red List (Kalkman et al., 2010; Riservato, Festi, et al., 2014). It is also listed in Annexes II and IV of the Habitats Directive (Cardoso, 2012) along with 16 other species of Odonata, making this taxonomic group the primary invertebrates in freshwater conservation (Dijkstra & Kalkman, 2012).

The aim of this study was to define the geographical boundaries of both species in Italy and to determine if they hybridized in the contact area. Based on the results, we discuss (a) the dynamics of postglacial expansion of the two species after the LGM, that led to the secondary contact between the taxa in central Italy; (b) the hybridization in relation to morphology and sexual selection; (c) the nature and the extension of the hybrid area within a conservation perspective.

## 2 MATERIALS AND METHODS

**Figure 1:**
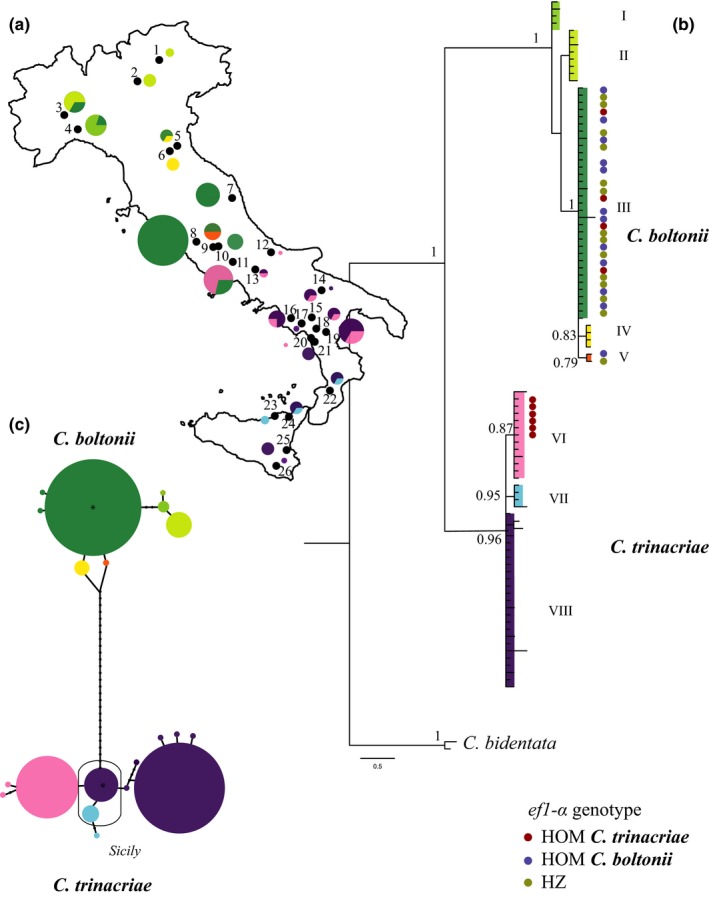
(a) Study area. The map reports the sampling localities (black dots, the numbers refer to the localities reported in Table 1) and the distributions and frequencies of the lineages (I–VIII in the *coI* tree) of *Cordulegaster boltonii* and *Cordulegaster trinacriae*. (b) Bayesian consensus tree for *coI*. Color bars and Roman numerals indicate the lineages within the two main clades representing the two species. The dots represent the intermediate individuals and the colors represent the nuclear assignment for each intermediate (see legend in the figure, Results, and Figure 2 for color details). (c) Parsimony networks performed on the *coI* data. The thin rectangle delimits the Sicilian haplogroups

In total, 93 male dragonflies belonging to the putative species *C*. *boltonii* and *C. trinacriae* were collected between 2008 and 2012 at 26 localities in the Italian peninsula (Table 1, Supporting Information Table S1 and Figure 1a). The specimens were preliminarily attributed to one of the above species according to the morphological characters given by Boudot (2001) and Dijkstra & Lewington (2006). For the sampling of *C. trinacriae* the Italian Ministry of the Environment issued a special permit (Prot DPN‐2009‐0017899). Twenty‐seven specimens clearly belonging to *C. boltonii* were sampled at seven localities in northern and central Italy and 36 specimens clearly belonging to *C. trinacriae* were collected at 14 localities in southern and central Italy. Thirty individuals from four localities in central Italy (localities 8‐11 Table 1 and Figure 1a) could not unequivocally be ascribed to either species, as the sexual appendages often presented intermediate shape (examples in the figures in Table 1). The left superior appendage and the inferior appendage of all individuals were photographed with a Hitachi TM1000 environmental scanning electron microscope (ESEM). Leg tissue samples of all individuals were collected for the DNA analysis and preserved in 100° ethanol.

**Table 1:**
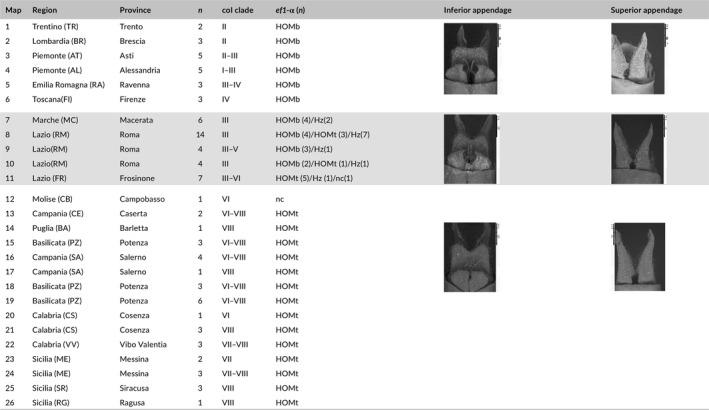
Sample details. Number of the locality on the map (*map*); sampling localities (*Region*,* Province*); number of individuals per locality (*n*); mitochondrial lineages present at each locality (*coI* clade); nuclear (*ef1*‐α) genotype for individuals at each locality and number of individual assigned to each category (*n*) when not all the individuals are assigned to the same one. In the column, the categories stand for HOMb: homozygous *C. boltonii*; HOMt: homozygous *C. trinacriae*; Hz: heterozygous. The columns *Inferior appendage* and *Superior appendage* show typical appendages for *C. boltonii* (localities 1–6); a hybrid (localities 7–11) and *C. trinacriae* (localities 12–26)

### 2.1 Molecular data analysis (i) Phylogeography, phylogeny and hybridization

The DNA was extracted from dissected metafemoral muscles of all 93 individuals and of two individuals of *C. bidentata* (used as outgroups in the phylogenetic analysis) according to the salting out procedure (Aljanabi & Martinez, 1997). Two markers were amplified: a fragment of the mitochondrial (mt) cytochrome c oxidase subunit I gene (*coI*) and the elongation factor 1α (*ef1*‐α) nuclear gene (nu). The mt DNA is a non recombining marker, easily tracked across nuclear backgrounds (Currat et al., 2008; Excoffier, Foll, & Petit, 2009; Petit & Excoffier, 2009) and therefore, useful to detect asymmetric introgression. The use of two markers with distinct modes of inheritance provides insight into the degree of reproductive isolation between lineages and lead into particular isolating mechanisms (Harrison & Bogdanowicz, 1997; Jiggins & Mallet, 2000; Wirtz, 1999), especially in terms of mate choice‐based introgression asymmetries. A 570 bp fragment at the 5′ terminus of the *coI* gene was amplified with the universal barcode primers LCO‐1490 and HCO‐2198 (Folmer, Black, Hoeh, Lutz, & Vrijenhoek, 1994) under standard PCR conditions. Both forward and reverse primers were used to sequence the fragment in double strand. For the *ef1*‐α*,* we amplified 550 bp using the primers EF1Fa and EF1Ra described in Pilgrim & Von Dohlen (2008). The PCR profile consisted of 35 cycles of 94C°/30 s denaturation, 54C°/60 s annealing and 72C°/60 s extension. All the sequences were inspected for double peaks, edited and aligned using Geneious v. 9 (Biomatter).

The Bayesian Inference (BI) approach was used to construct the *coI* phylogenetic trees. A Generalized Time‐Reversible model with a proportion of invariable sites and heterogeneous substitution rates following a gamma distribution (GTR + I + G, Rodríguez, Oliver, Marín, & Medina, 1990) was selected as the best substitution model under the AIC criterion as implemented in JModelTest (Posada, 2008). The Bayesian phylogenetic analysis was performed under the selected substitution model and was carried out with MrBayes v. 3.2.1 (Huelsenbeck & Ronquist, 2001) by running 1,000,000 generations, with Markov chains sampled every 1000 generations. The convergence of the parameters was determined with the software TRACER 1.6 (Rambaut, Suchard, Xie, & Drummond, 2014). A burn‐in of 10% was applied and the remaining trees were used to compute a 50% majority rule consensus tree and posterior probabilities. To reconstruct *coI* phylogeography within the two species we built a parsimony network on the entire *coI* dataset with TCS v. 1.21 (Clement, Posada, & Crandall, 2000), in default settings, calculating connection limits at 30 steps. The time of the most recent common ancestor (TMRCA) was estimated using BEAST v. 1.8 (Drummond, Suchard, Xie, & Rambaut, 2012). Given the lack of fossil records for Cordulegastridae and its related families Chlorogomphidae and Neopetalidae, as calibration points we used an average value of the *coI* substitution rate in a range between 1.5% and 2.3%, values estimated for mitochondrial DNA in insects (Brower, 1994; Farrell, 2001; Quek, Itino, & Pierce, 2004). Thus, we applied a lognormal distribution (*μ* = 0.019; *SD* = 0.15) to include the maximum and minimum substitution rate values. The analysis was performed three times independently, with 100 million generations and sampling of trees every 10,000 steps using a Yule process as tree model. TRACER 1.6 (Rambaut et al., 2014) was used in all the BEAST analyses to check for parameter convergences and stationarity.

Because of the presence of multi‐site heterozygotes in the alignment of the nuclear *ef1*‐α gene (see Results), the gametic phase of each genotype was resolved using PHASE (Garrick, Sunnucks, & Dyer, 2010; Stephens & Donnelly, 2003; Stephens, Smith, & Donnelly, 2001) implemented in DNASp v. 5 (Librado & Rozas, 2009). Three independent runs of 1,000 iterations were performed with the initial 1,000 iterations discarded as burn‐in and one as thinning interval. To evaluate the relations among the nuclear haplotypes, we used the phased haplotypes to construct a parsimony network with TCS v. 1.21 (Clement et al., 2000), in default settings, calculating connection limits at 95%. To estimate gene flow patterns between *C. boltonii* and *C. trinacriae*, we used the Isolation‐with‐Migration model as implemented in IMa2 (Hey, 2010). The two‐populations analysis was conducted constraining the nuclear DNA dataset according to the mtDNA information and therefore, treating the hybrids on the basis of their respective mtDNA assignment. Preliminary short runs were conducted to determine the most appropriate upper bound in order to incorporate the posterior distribution for each parameter. After obtaining the fitting scalars (‐t15, ‐q30, ‐m3), we performed a final M mode analysis using the HKY model for both genes, with 20 independent Markov‐coupled chains with a geometric heating scheme (‐ha0.96 and ‐hb0.9) as suggested for small to medium‐sized data sets (Hey, 2011). In total, 100,000 genealogies were retained and mixing properties among chains were checked by inspection of low parameter autocorrelations, high swap rates and no trend in the splitting time plots (Hey, 2010).

### 2.2 Molecular data analysis (ii) Demography and spatial analysis

The mode and time of population size changes for both species were inferred using the multilocus Extended Bayesian Skyline Plot (EBSP, Drummond et al., 2012). To provide the most reliable demographic reconstruction for each species, the analysis was carried out only on individuals identified as *C. boltonii* or *C. trinacriae* by both molecular markers, therefore, we excluded the hybrid specimens from the analysis. The final run was carried out with substitution models, clock rates and tree models for the two genes kept unlinked. At last, we performed a Bayesian phylogeographic reconstruction in continuous space (Lemey, Rambaut, Welch, & Suchard, 2010) to identify the ancestral areas and from them the process of spatial diffusion. After fine‐tuning the prior parameters, we ran a final analysis using a relaxed random walk diffusion model (RRWs) with Cauchy distribution (Lemey et al., 2010). Each species was processed separately with application of a strict clock model and a Gaussian Markov Random Field (GMRF) Bayesian Skyride (Minin, Bloomquis, & Suchard, 2008) as a coalescent tree prior. After computing the MCC with TreeAnnotator 1.7.5, we visualized the processes of spatial diffusion from ancestral areas using the Time Slicer option in SPREAD v. 1.0.5 (Bielejec, Rambaut, Suchard, & Lemey, 2011). We performed both the demographic inference and the spatial diffusion reconstruction in BEAST v. 1.7 (Drummond et al., 2012), running 300 million generations, sampling parameters every 30,000 generations and providing the *coI* substitution rate setting as previously described for the TMRCA analysis.

### 2.3 Geometric morphometrics

**Figure 2:**
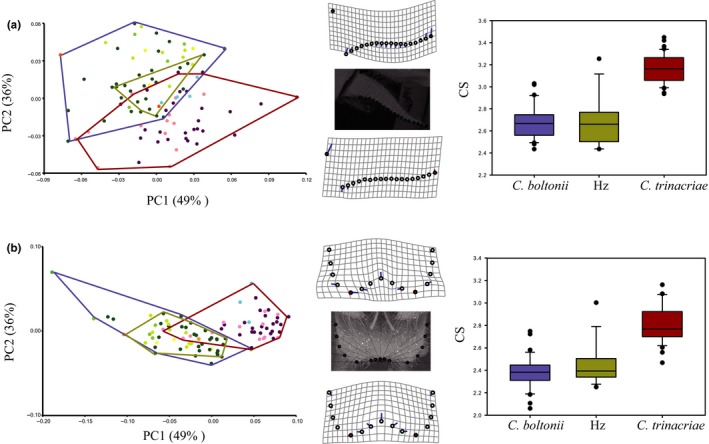
Geometric morphometrics analysis. (a) Superior appendage. (b) Inferior appendage. From left to right in (a) and in (b): Principal component analysis scatter plot (the percentage of explained variance is reported between parentheses) showing shape variability of *Cordulegaster boltonii* (blue polygon), *Cordulegaster trinacriae* (red polygon) and hybrid individuals (yellow polygon); Collected landmarks (see Materials and Methods for details); Deformation grids; Comparison of centroid size, the inner line is the median, the box margins are the 25th and 75th percentiles, bars extend to the 5th and to the 95th percentiles

The two diagnostic characters, the inferior appendage and the left superior appendage, were photographed with a Hitachi TM1000 environmental scanning electron microscope (ESEM) for all 93 individuals (example figures in Figure 2 and in Table 1). The form of these appendages is used to distinguish the two species (Boudot, 2001; Dijkstra & Lewington, 2006). In male Odonata they interlock with parts of the female body during tandem formation (Corbet, 1999) and can be mechanically incompatible with sister species resulting in and efficient prezygotic isolation mechanism (Monetti et al., 2002). When the left superior appendage was damaged or missing, a mirror image of the right superior appendage was used. The geometric morphometrics approach, based on the Procrustes method (Bookstein, 1991), was used to analyze the morphological inter‐intraspecific variation. To define the position of the landmarks on curved structures in an unambiguous way, we superimposed a “comb” on each image. The extremities of the comb were positioned at homologous points (included as landmarks in the analysis) and the semi‐landmarks were selected at the intersection between the teeth of the comb and the margin of the structure (Figure 2). Fifteen landmarks were digitized on the inferior appendage and 19 landmarks on the superior appendage using tpsDig2 (Rohlf, 2006). We had planned to investigate the form of the entire left superior appendage, but the inner margin proved to be highly variable in response to slight differences in orientation, and therefore only the outer margin was digitized. A Generalized Procrustes Analysis (GPA, Rohlf & Slice, 1990) was performed to convert the landmarks into separate sets of variables for size (centroid size, CS) and shape (Procrustes coordinates).

The overall shape variability was analyzed by Principal Component Analysis (PCA) and graphically evaluated through deformation grids.

A second PCA analysis was performed on a subset of individuals identified as *C. boltonii* and *C. trinacriae* by both molecular markers (see Table 1, Supporting Information Table S1). Canonical Variates Analysis (CVA) with a permutation test was applied to the same data set to test the presence of significant shape differences between the two species [evaluated by Multivariate Analysis of Variance (MANOVA)]. Size differences among *C. boltonii*,* C. trinacriae,* and hybrid individuals were investigated by ANOVA (Tukey test) performed on the CS and graphically represented by a box‐plot. A regression between the shape variables and CS was carried out to investigate the influence of size on shape. At last, we carried out a regression of Procrustes coordinates on the latitude values to test if the morphological variability depended on geographical/ecological factors. In fact, the distribution of both species covers a large latitudinal range and we found that the differences in the shape of appendages were driven by the differences in size (see Results). Therefore, it was important to test whether this variation in shape depends on ecological factors, as size is known to be influenced by latitude in Odonata (Johansson, 2003; Sniegula, Golab, & Johansson, 2016). The analyses were carried out on the inferior and superior appendages separately with the software MorphoJ 1.06b (Klingenberg, 2011), with the exception of ANOVA performed with PAST 3.11 (Hammer, Harper, & Ryanet, 2001) and MANOVA performed in R (R Development Core Team, 2011).

## 3 RESULTS

The 111 new sequences (21 *coI* haplotypes and 90 *ef1*‐α genotypes) of Italian *Cordulegaster* are deposited in GenBank, Accession Numbers MH304646 ‐ MH304756 (see Supporting Information Table S1 for details).

The Bayesian consensus tree for *coI* is reported in Figure 1b (only posterior probability (*p.p*.) values exceeding 70% are shown). The estimated ESS values were greater than 200 for all parameters. There are two highly supported principal clades, corresponding to the two species *C. boltonii* (*p.p*. = 1) and *C. trinacriae* (*p.p*. = 0.96). The first clade comprises individuals from northern and central Italy, including those morphologically identified as *C. boltonii*, while the second clade includes individuals from southern and central Italy, morphologically *C. trinacriae*. The analysis identified five lineages (I–V) in *C. boltonii* and three lineages (VI–VIII) in *C. trinacriae*. The specimens with intermediate morphology fall into both clades. In the *C. trinacriae* clade, six of these individuals from central localities (11 and 12) are in lineage VIII (*p.p*. = 0.87). In the *C. boltonii* clade, we found 24 intermediate individuals: 22 are in the highly supported lineage III (*p.p*. = 1), and two are in the lineage V (*p.p*. = 0.79).

The parsimony network performed on the two *coI* clades is reported in Figure 1c. In *C. boltonii*, two haplogroups can be distinguished: the larger one contains the haplotypes from northern and central Italy (5 haplotypes) and the other contains all the other northern haplotypes (3 haplotypes). In *C. trinacriae*, three distinct haplogroups can be distinguished and the Sicilian haplotypes constitute the core of the network.

According to our estimate of the TMRCA, the split between *C. trinacriae* and C*. boltonii* occurred about 1.32 Mya [95% highest posterior density (HPD): 0.56–2.17, early Pleistocene].

**Figure 3:**
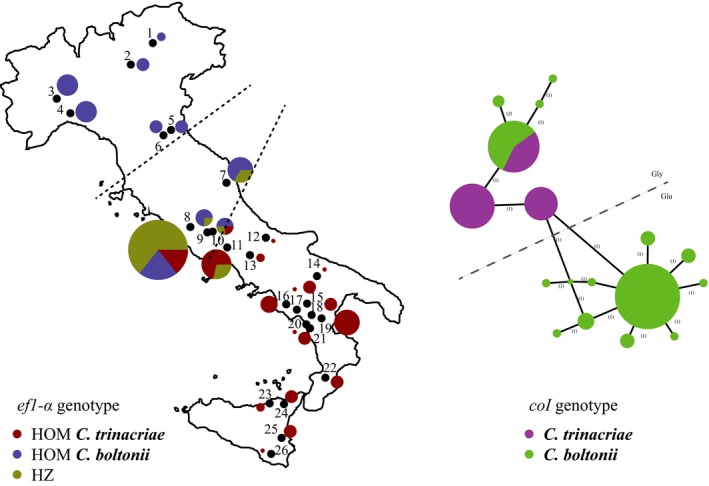
Nuclear marker analysis. Map of the geographical distribution of *ef1*‐α genotypes (left) and parsimony network on the phased haplotypes (right), colored following the *coI* clades. The dashed lines on the map indicate the approximate extension of the hybrid area (see Results). The mutation producing the amino acid substitution between glycine (Gly) and glutamic acid (Glu) is indicated by a dashed line on the parsimony network

The nuclear *ef1*‐α analysis resulted in multi‐site heterozygote sequences, with 12 positions characterized by double peaks. Several individuals present a polymorphism in three of these heterozygous positions (positions 186, 377 and 523 on the 550 bp *ef1*‐α alignment). The heterozygous sequences are distributed only among the intermediate individuals. The only exceptions are two of the six individuals from locality 7, Macerata (MC.SaG.3 and MC.SaG.4; Table 1 and Supporting Information Table S1; Figure 1), identified both morphologically and mitochondrially as C*. boltonii* but polymorphic for *ef1*‐α. Eleven mutations are in the third codon position and they are silent. Instead, the substitution in position 377 of the alignment is in the second codon position and it changes the codon from GGG to GAG, producing an amino acid change in the protein sequence from glycine (Gly) to glutamic acid (Glu). The parsimony network on the phased haplotypes (Figure 3) resulted in two main haplogroups corresponding to south‐central Italy and north‐central Italy, respectively, and thus identifying the two species *C. trinacriae* and *C. boltonii*. The two groups are separated by the mutation that produced the amino acid substitution (Figure 3). Therefore, this mutation was used to assign the individuals to the two species and to identify the hybrids. In the heterozygotic specimens, we found two different conditions: (a) both phased haplotypes of an individual fall in the same haplogroup and, in this condition, we assigned the individual (homozygous) to the corresponding species; (b) the two‐phased haplotypes fall into two different haplogroups and, in this condition, we define an individual as hybrid. At last, we found that all the morphological intermediate individuals assigned mitochondrially to *C. trinacriae* were attributed to the same species with the nuclear marker. In contrast, for the intermediate individuals assigned mitochondrially to *C. boltonii* the nuclear marker returned three different conditions: (a) *C. boltonii* genotype (*n* = 9); (b) hybrid genotype (*n* = 14); (c) *C. trinacriae* (introgressed) genotype (*n* = 4). These hybrids also include the two individuals from Macerata which were morphologically attributed to *C. boltonii* (tree in Figures 1 and 3; Table 1).

**Figure 4:**
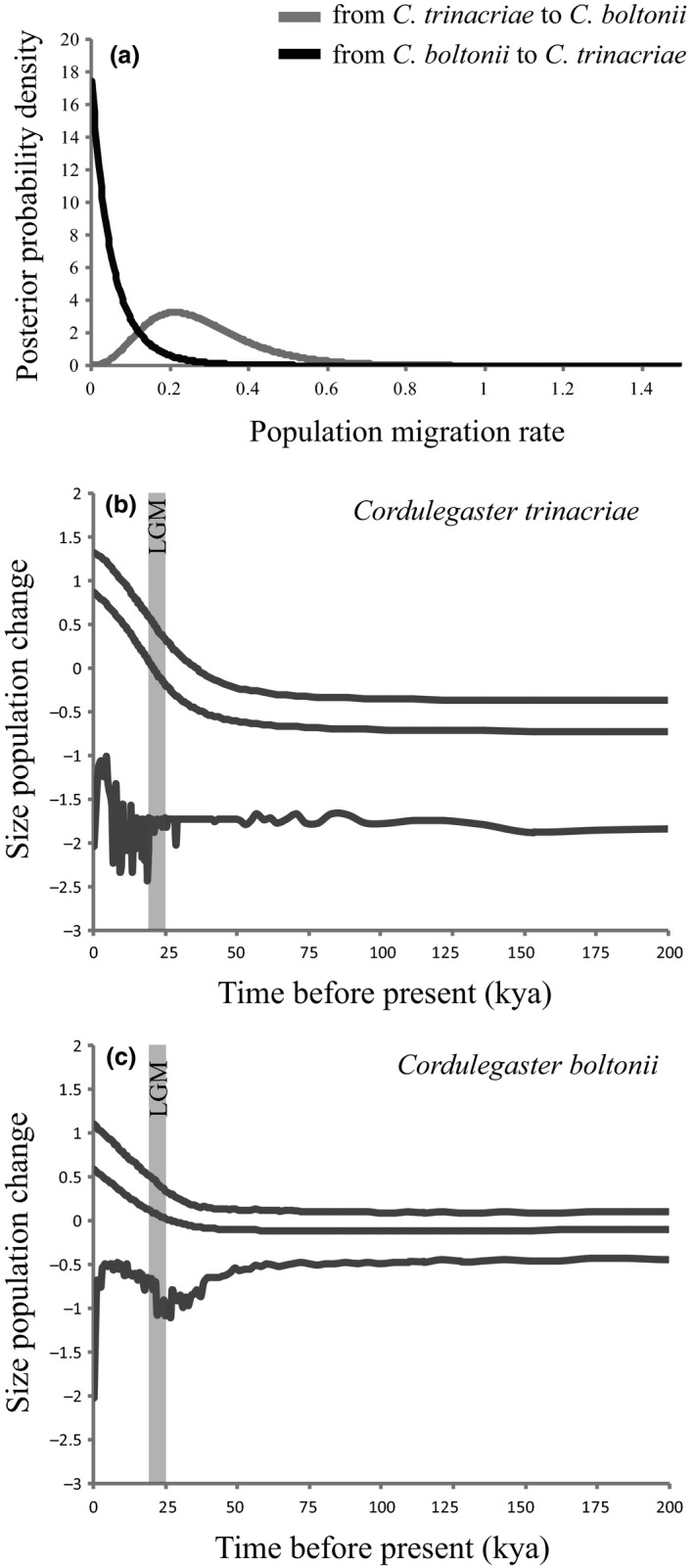
(a) Marginal posterior densities for the migration rate between *Cordulegaster boltonii* and *Cordulegaster trinacriae* inferred using IMa2, (b) and (c) Extended Bayesian Skyline Plot (EBSP) representing the historical demographic trend. The vertical gray bar indicates the Last Glacial Maximum (LGM)

**Figure 5:**
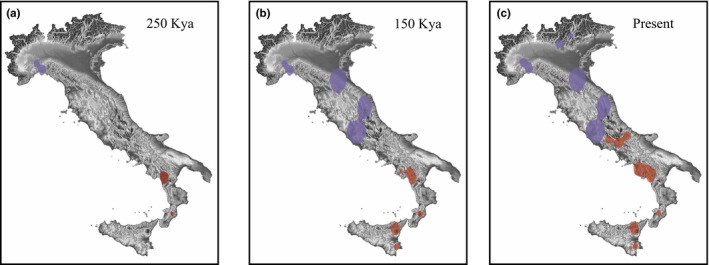
Reconstruction of the spatial diffusion process at three different time slices: (a) 250,000 ka; (b) 150,000 ka; (c) present. The colors of the polygons (representing 80% HPD uncertainty) refer to the two species: in blue *Cordulegaster boltonii* and in red *Cordulegaster trinacriae* populations

The migration parameters estimated using the IMa2 analysis reveal significant gene flow from *C. trinacriae* to *C. boltonii* but not vice versa (*m*_Ct_ = 0.281, 95% HPD = 0.05–0.53; *m*_Cb_ = 0.063, 95% HPD = na‐0.1; LLR = 10.698, *p* < 0.001). The marginal posterior densities for the migration rate between the two species are reported in Figure 4a. The EBSP (Figure 4b,c) produced informative coalescent intervals from 200,000 ya. From this point in time, the demographic trend remained stable up to the LGM when population growth occurred simultaneously in both species. *Cordulegaster boltonii* showed slow population growth, whereas *C. trinacriae* exhibited fast population growth, with a doubling in population size with respect to *C. boltonii* in the same time span. The Bayesian phylogeographic reconstruction performed on *coI* (Figure 5a,b,c) placed the ancestral populations of *C. boltonii* in the north‐western part of Italy (localities 3 and 4). From there, the species expanded toward the center and then faster toward the northeast. Two ancestral areas are evident in *C. trinacriae*, the first placed in Basilicata, Campania and northern part of Calabria (localities 14‐ 21), the second in the southern part of the same region. The colonization moved southward and then upward to central Italy. The analysis showed that *C. boltonii* was present in central Italy by 150,000 ya (Figure 5a), whereas the arrival of *C. trinacriae* was a more recent event (Figure 5c).

### 3.1 Geometric morphometrics

The PCA of the superior appendage is reported in Figure 2a. The PC1 vs. PC2 plot explains 85% of the shape variability and shows the presence of two distinct groups separated along both axes, which, however, partially overlap. The bottom group contains only individuals assigned to *C. trinacriae* based on the molecular data (*coI* and *ef1*‐α). The upper group consists of all individuals molecularly assigned to *C. boltonii* and all the hybrid specimens. The individuals of the two nominal species are morphologically well distinguished and the intermediate individuals are situated in the overlap area. The deformation grids (Figure 2) showed that the shape differences between the species are mainly due to a more sinuous outer margin and to a greater overall length of the appendages in *C. trinacriae*.

The PCA of the inferior appendage is reported in Figure 2b. Also, in this case, the PC1 vs. PC2 plot explains 85% of overall shape variability and shows results similar to those of the superior appendage. Again, there are two distinct groups separated along PC1 (49% of variability explained), the left one contains only individuals molecularly assigned to *C. trinacriae* and, the right group contains the individuals molecularly assigned to *C. boltonii* and the hybrids. The intermediate individuals are situated in the overlap area. The deformation grids (Figure 2) showed that the shape changes are located at the hind margin of the inferior appendages, which are deeply notched in *C. trinacriae*, and at the side margins which in *C. trinacriae* are more parallel to the hind margins.

The analyses (performed separately on the superior and inferior appendages) of a sub‐data set containing only those specimens molecularly assigned to *C. trinacriae* and *C. boltonii* with both molecular markers revealed two well distinct groups on the PCA (not shown). The CVA carried out on this sub‐data set showed that the two groups corresponding to the two species are morphologically significantly different [MANOVA: Wilks’ *λ*_sup.app._ = 7.784E‐120, *p* = 0; Wilks’ *λ*_inf.app_ = 7.289E‐155, *p* < 0.0001].

The analysis of size of both the superior and inferior appendages (Figure 2a,b) confirmed a significant difference between *C. trinacriae* and *C. boltonii* [inferior appendage: Tukey test *p* < 0.0001; superior appendage: Tukey test *p* < 0.0001]. The superior appendage of *C. trinacriae* is significantly longer and the inferior appendage is significantly wider than those of *C. boltonii*. Hybrid individuals have an intermediate size and are not significantly different from *C. boltonii* [inferior appendage: Tukey test *p* = 0.4340; superior appendage: Tukey test *p* = 0.9977] but significantly different from *C. trinacriae* [inferior appendage: Tukey test *p* < 0.0001; superior appendage: Tukey test *p* < 0.0001].

The regression of the Procrustes coordinates on the CS, for both superior and inferior appendages, showed a correlation between the two components [inferior appendage: 34.8% predicted/*p* < 0.0001; superior appendage: 22.97% predicted/*p* < 0.0001], highlighting the presence of an allometric factor in differentiating the appendages of the species. According to that the PCA performed on the residuals of the regression showed no differentiation between the two species (data not shown). The plot of the PCA performed separately on each species was not correlated to the geographical distribution of the individuals. In a similar way, the regression between the Procrustes coordinates (calculated separately for the two species for superior and inferior appendages) and latitude showed no evidence of correlation [inferior appendage: *C. boltonii* vs latitude: 5.77% predicted/*p* = 0.1463; *C. trinacriae* vs latitude: 60.2% predicted/*p* = 0.8337; superior appendage: *C. boltonii* vs latitude: 20.34% predicted/*p* = 0.0008; *C. trinacriae* vs latitude: 11.55% predicted/*p* = 0.0104].

## 4 DISCUSSION

### 4.1 Preliminary overview

Emerging evidence reveals that postglacial routes of animals and plants in the Italian Peninsula followed complex species‐specific patterns (Chiocchio, Bisconti, Zampiglia, Nascetti, & Canestrelli, 2017; Mouton et al., 2012; Provan & Bennett, 2008; Schmitt, 2007). The common trend is that northward range shifts initiated from southern refugia (Hewitt, 1999, 2000, 2001; Taberlet et al., 1998). In contrast, for *Cordulegaster* we found that the two species survived in distinct refugia, two located in the south (*C. trinacriae*) and one in the north (*C. boltonii*), and then expanded until they reached a secondary contact zone in central Italy where they hybridized.

*Cordulegaster boltonii* in the north and *C. trinacriae* in the south occupy a large part of Italy (Riservato, Festi, et al., 2014). However, the limits of their distributions were unknown, as was the area where the two species came into contact (Galletti & Pavesi, 1985). Individuals from the north are morphologically distinguishable from those from the south (Boudot, 2001; Dijkstra & Lewington, 2006), but we found that several specimens from central Italy show intermediate characters and this was the first empirical evidence of a possible hybridization between the two taxa.

The mitochondrial molecular analysis (Figure 1) revealed two distinct clades corresponding to the geographical distribution of the two species and thus, we attributed individuals of the north‐central clade to *C. boltonii* and those of the south‐central clade to *C. trinacriae*. Individuals with an intermediate morphology were found within both of mitochondrial clades.

The analysis of the nuclear marker (Figure 3) revealed the presence of heterozygosity only in individuals with an intermediate morphology, with two exceptions represented by the individuals from Marche (locality 7). Although there are three main heterozygous positions in *ef1*‐α, only the amino acid substitution is diagnostic for hybrids (network in Figure 3); hybrids were identified from localities 7, 8, 9, 10 and 11. When the *ef1*‐α heterozygosity pattern is combined with the mitochondrial outcome (tree in Figure 1b), all specimens of the *coI* clade of *C. trinacriae* were homozygous for the *ef1*‐α. In contrast, all the conditions of homozygosity and heterozygosity of the nuclear marker were found in individuals mitochondrially assigned to *C. boltonii*. This indicates that: (a) the two species hybridize; (b) the hybrid individuals carry only one type of mitochondrial DNA (the *C. boltonii* type), indicating that *C. boltonii* females mate interspecifically and intraspecifically; (c) therefore, the gene flow is asymmetric and directed from *C. trinacriae* to *C. boltonii*.

### 4.2 Demography and postglacial expansion

*Cordulegaster trinacriae* and *C. boltonii* have an early Pleistocene origin, in the Calabrian age. Their separation is dated at ca. 1.32 Mya. Considering *C. boltonii* having a Palaearctic distribution, this datum indicates a split from C*. trinacriae* in the Pleistocene and may reflect the geomorphological situation of the Italian Peninsula during this period. In fact, during the early Pleistocene the Italian Peninsula was characterized by a series of environmental discontinuities due to the presence of the Apennines and primed by repeated glacioeustatic sea‐level fluctuations which isolated the populations along the west–east axis (Cremaschi, 2003a,b; Di Giovanni, Vlach, Giangiuliani, Goretti, & Torricelli, 1998; Giraudi, 2004; Stefani, Galli, Crosa, Zaccara, & Calamari, 2004).

The demographic analysis showed exponential growth of the populations of both species at the end of the LGM (Figure 4b,c). The beginning of population expansion (dated between 50,000 and 25,000 ya) coincides with the decline of the Ice Age and thus is a typical expansion following the postglacial ice retreat (Hewitt, 1999, 2000, 2001). Over the same time span, *C. trinacriae* showed a more rapid increase in population size, twice that of *C. boltonii* (Figure 4b,c). This recent expansion may also have been responsible for the slightly higher population variability (Figure 1a) detected with the mitochondrial marker.

The spatial diffusion analysis revealed three main ancestral areas (Figure 4d–f), one in northern Italy (*C. boltonii*) and two in southern Italy (*C. trinacriae*). From these areas, there were two chronologically different dispersal for the two species. *Cordulegaster boltonii* reached its current distribution before the beginning of the last glacial age (approximately 150,000 ya) and remained at a low population density until slightly increasing in proximity to the LGM, as inferred from the demographic analysis (Figure 4b). In contrast, *C. trinacriae* underwent a sudden demographic and spatial expansion from the ancestral areas approximately around the LGM (Figures 4c, 5).

From its refugium in the north‐western sub‐Alpine region, *C. boltonii* expanded first to the center and later to north‐eastern Italy, accordingly to the presence of other xerothermic micro‐refugia located south of the Alps (Bhagwat & Willis, 2008; Kaltenrieder et al., 2009). For *C. trinacriae*, the spatial diffusion analysis (Figure 5a‐c) supported the presence of two ancestral distributions, the first located between Campania, Basilicata and Calabria and the second in southern Calabria (Figure 5a). From the two refugia, the colonization proceeded first southward and then toward the center. The spatial data suggest that *C. boltonii* was present in central Italy earlier than *C. trinacriae*, indicating a recent contact between the two species.

### 4.3 Asymmetric hybridization and morphological constraints

The Ima2 analysis (Figure 4a) revealed the presence of asymmetric gene flow from *C. trinacriae* to *C. boltonii*. Unidirectional hybridization has also been observed in the Odonata genera *Enallagma* and *Ischnura* (Sánchez‐Guillén et al., 2011; Turgeon et al., 2005), and for *Enallagma* it was shown that male sexual appendages are primarily used to identify potential mates (McPeek, Shen, Torrey, & Farid, 2008). The reproductive isolation in damselflies is complex because it is caused by multiple mechanisms, including both premating and postmating barriers (Wellenreuther & Sánchez‐Guillén, 2016). Often, in Odonata, the structures involved in mating can act as a premating barrier, resulting in morphological incompatibility during tandem formation and copulation (Monetti et al., 2002; Paulson, 1974; Sánchez‐Guillén, Wellenreuther, & Cordero Rivera, 2012; Wellenreuther & Sánchez‐Guillén, 2016). In the present study, we found evidence that the two species are morphologically well characterized in regard to superior and inferior appendages (Figure 2 and MANOVA). It is interesting that the hybrid individuals fall exclusively within the range of variability of *C. boltonii*. The morphological differences between the two species are not confined to shape, indeed the appendages of *C. boltonii* and those of the hybrid individuals are significantly smaller than those of *C. trinacriae* (box plots in Figure 2a,b). The analysis of allometry suggests that the change in appendages shape was driven by differences in their size. In keeping with this hypothesis, no correlation of shape with latitude was found. This excludes geographical/ecological causes of the differences in shape and confirms the genetic bases of the appendages morphology.

The smaller appendages of the *C. boltonii* males might be responsible for a reduced ability to mate with *C. trinacriae* females. Vice versa, the longer appendages of the *C. trinacriae* males may allow for mating with the females of both *C. boltonii* and *C. trinacriae*. If this hypothesis is correct, these morphological differences may have a role in determining the direction of hybridization between the two species. The genetic data support this idea and are in accordance with the fact that interspecific mating is prevented mainly in one direction and, consequently, there is only limited and asymmetric prezygotic reproductive isolation. Several previous studies indicated this effect in Odonata. In fact, a similar condition was observed by Sánchez‐Guillén et al. (2011) between two sister species of *Ischnura* and in laboratory experiments by Monetti et al. (2002). The small males of *I. graellsii* cannot grasp the protothorax of the large *I. elegans* females, and this proves to be a very efficient isolation mechanism. Our results show that *C. boltonii* reached central Italy first and *C. trinacriae* arrived later, after moving northward (Figure 5a,b,c). Furthermore, the data show that *C. trinacriae* was the species with faster population growth, invading the other one in the contact area (Figure 3). In the perspective of neutral gene introgression during expansion, the direction of hybridization is supposed to be the opposite (Currat et al., 2008), that is, from the local (*C. boltonii*) to the invading species (*C. trinacriae*). On the contrary, in the perspective of sexual selection (Wirtz, 1999) the invading females can mate with both local and immigrant males to enhance their fitness, that is, from the invading females (*C. trinacriae*) to the local species males (*C. boltonii*). In our case, we found introgression from the invading males (*C. trinacriae*) to the local females (*C. boltonii*). In fact, *C. trinacriae* is the advancing species but it has longer sexual appendages. Hence, those findings may suggest a role of sexual appendages size in constrain the direction of intraspecific matings (Wirtz, 1999), as seen also in Sánchez‐Guillén et al. (2011). Experimental work on these *Cordulegaster* species would be necessary to ultimately confirm the effectiveness of this reproductive barrier.

### 4.4 Hybrid area extension and conservation implications

The geographical distribution of nuclear haplotypes (Figures 1b and 3) indicates that the hybridization covers an extensive area stretching the entire width of the central Italian Peninsula. Such a wide‐ranging introgression has also been observed in another species of Odonata (Hayashi et al., 2005). The hybrid zone extends from Marche to southern Latium (localities 7–11). We can set Marche as the northern boundary because of the presence of the two individuals (MC.SaG.3 and MC.SaG.4, locality 7) which are mitochondrially and morphologically *C. boltonii* whereas their nuclear genome was found to be hybrid, probably of a generation following the F1. In the case of secondary contact, it is important if one of the species involved is endangered, in which case it is necessary to establish contingent consequences for its conservation and to formulate specific management policies (Genovart, 2009).

Climate change has been shown to drive changes in phenology and distribution of several animal taxa (e.g., butterflies Parmesan et al., 1999; Parmesan, 2006; Bellard, Bertelsmeier, Leadley, Thuiller, & Courchamp, 2012) including odonates (Hassall, Thompson, & French, 2007; Hickling, Roy, Hill, & Thomas, 2005; Sánchez‐Guillén et al., 2011, 2014). Sánchez‐Guillén et al. (2011) showed that *Ischnura elegans*, became sympatric to its sister species changing its phenology in a relatively short time span and then almost completely replacing it through unidirectional hybridization. The authors showed that climate change could be a factor driving directly the described events (Sánchez‐Guillén, Muñoz, Rodríguez‐Tapia, Feria Arroyo, & Córdoba‐Aguilar, 2013). Climate change can trigger hybridization in several ways, for example, by population range shifts due to habitat loss (Rhymer & Simberloff, 1996; Sánchez‐Guillén et al., 2011; Taylor, Larson, & Harrison, 2015) or/and by environmental changes favouring hybrids over parental populations in the hybrid zones (see examples in the review by Seehausen, 2006). In any case, these events can have a strong impact in loss of diversity, with consequent conservation implications (Rhymer & Simberloff, 1996; Sánchez‐Guillén et al., 2011; Seehausen, 2006).

Our results clearly indicate that *C. trinacriae* has been moving northward in the last few thousand years (Figure 5a,b,c), long before the onset of climate change, and this southern species is intruding into *C. boltonii*'s territory. The direction of the range shift is essential from a conservation point of view, as *C. trinacriae*, the expanding taxon, is an endangered and protected species (see above).

When a rapidly‐expanding species invades the territory of a sister species creating well‐adapted hybrids, the hybridization area may be destined to be stable and might even extend further. A plausible outcome could be disappearance of one or both of the parental species, with a tendency toward the mixing of the genomes and the spreading of intermediate morphological characters along the entire peninsula (Sánchez‐Guillén et al., 2014; Wolf et al., 2001). From a conservation point of view, even if *C. trinacriae* currently has a strong genetic identity, the presence of extended hybridization between the two species, should be considered relevant for future species management.

## 5 CONCLUSIONS

This study assessed the presence of asymmetric hybridization after postglacial secondary contact between the two sister species and is in line with the results of recent studies on various animal species showing that the postglacial patterns can show numerous facets (i.e., Alpine xerothermic refuge), even though they follow the general dynamics described by Hewitt (2000).

The use of two loci over the entire genome imposed clear limit to our study. The use of several unlinked genes or even better of genome‐wide loci is highly desirable to have the most accurate inference about hybridization and demography. However, the present study raises interesting questions for future and experimental works. In fact, studies on local dispersal in Odonata indicate that the animal's wing size and load are related to mobility, and these aspects are predictive of their flight ability (Conrad et al., 2002; Grabow & Ruppell, 1995). Therefore, if asymmetric hybridization due to sexual preference, is linked to dispersal propensity (i.e., dispersal syndrome, Travis & Dytham, 2002; Shine, Brown, & Phillips, 2011), it can be hypothesized and tested in future, that the mating of *C. trinacriae* males—with a strong propensity for dispersal—with females of both species could be the outcome of spatial sorting of multiple traits evolved during the range expansion.

At last, as the larvae of the *Cordulegaster* species live in vulnerable habitats, they can be an excellent model to study the effects of climate change on biodiversity and on the fate of these particularly stenotopic species.

## CONFLICT OF INTEREST

None declared.

## AUTHOR CONTRIBUTION

Dr. Emanuela Solano contributed to the acquisition, analysis, and interpretation of data. The author drafted the manuscript and all the revision of the co‐authors. Dr. Sönke Hardersen contributed to the acquisition and interpretation of data, drafted and revised all manuscript versions. Prof. Paolo Audisio contributed to the interpretation of data and revised all manuscript versions. Dr. Valentina Amorosi contributed to the acquisition, analysis, and interpretation of data. Dr Gabriele Senczuk contributed to the analysis and interpretation of data, drafted part of the manuscript and revised all the versions. Dr. Gloria Antonini contributed to the data acquisition, analysis, and interpretation and coordinated the work of all co‐authors.

## DATA ACCESSIBILITY

The *coI* and *ef1*‐α sequences are deposited in GenBank, Accession Numbers MH304646 ‐ MH304756.
