# Supplementary material for: Asymmetric hybridization in Cordulegaster (Odonata: Cordulegastridae): Secondary postglacial contact and the possible role of mechanical constraints
Source: Ecol Evol. 2018 Sep 14;8(19):9657–71. doi: 10.1002/ece3.4368 (PMC6202705; doi:10.1002/ece3.4368)
Supplement: Supplementary file 1 [file ECE3-8-9657-s001.docx]

| Locality | | | map | n | Voucher | coI | ef-α | Field | CoI hp | coI AN | ef-α AN |
| --- | --- | --- | --- | --- | --- | --- | --- | --- | --- | --- | --- |
| Trentino | Trento | Baselga di Pinè, Rigagnolo | 1 | 2 | TR.Bas.1 | b | b | b | Cor2 | MH304647 | MH304667 |
|  |  |  |  |  | TR.Bas.2 | b | b | b | Cor2 | MH304647 | MH304668 |
| Lombardia | Brescia | Vobarno, Cecino | 2 | 3 | BS.Vob.1 | b | b | b | Cor2 | MH304647 | MH304669 |
|  |  |  |  |  | BS.Vob.2 | b | b | b | Cor2 | MH304647 | MH304670 |
|  |  |  |  |  | BS.Vob.3 | b | b | b | Cor2 | MH304647 | MH304671 |
| Piemonte | Asti | Rochetta Tanaro, Rio Rabengo | 3 | 5 | AT.RoT.1 | b | b | b | Cor1 | MH304646 | MH304672 |
|  |  |  |  |  | AT.RoT.2 | b | b | b | Cor2 | MH304647 | MH304673 |
|  |  |  |  |  | AT.RoT.3 | b | b | b | Cor2 | MH304647 | MH304674 |
|  |  |  |  |  | AT.RoT.4 | b | b | b | Cor1 | MH304646 | MH304675 |
|  |  |  |  |  | AT.RoT.5 | b | b | b | Cor2 | MH304647 | MH304676 |
| Piemonte | Alessandria | Bosio, Cascina Alberghi | 4 | 5 | AL.Bos.1 | b | b | b | Cor1 | MH304646 | MH304677 |
|  |  |  |  |  | AL.Bos.2 | b | b | b | Cor3 | MH304648 | MH304678 |
|  |  |  |  |  | AL.Bos.3 | b | b | b | Cor4 | MH304649 | MH304679 |
|  |  |  |  |  | AL.Bos.4 | b | b | b | Cor3 | MH304648 | MH304680 |
|  |  |  |  |  | AL.Bos.5 | b | b | b | Cor3 | MH304648 | MH304681 |
| Emilia Romagna | Ravenna | Rio Tanaccia | 5 | 3 | RA.Bri.1 | b | b | b | Cor5 | MH304650 | MH304682 |
|  |  |  |  |  | RA.Bri.2 | b | b | b | Cor1 | MH304646 | MH304683 |
|  |  |  |  |  | RA.Bri.3 | b | b | b | Cor1 | MH304646 | MH304684 |
| Toscana | Firenze | Bibbiana | 6 | 3 | FI.Bib.1 | b | b | b | Cor5 | MH304650 | MH304685 |
|  |  |  |  |  | FI.Bib.2 | b | b | b | Cor5 | MH304650 | MH304686 |
|  |  |  |  |  | FI.Bib.3 | b | b | b | Cor5 | MH304650 | MH304687 |
| Marche | Macerata | San Ginesio, Landi | 7 | 6 | MC.SaG.1 | b | b | b | Cor1 | MH304646 | MH304688 |
|  |  |  |  |  | MC.SaG.2 | b | b | b | Cor1 | MH304646 | MH304689 |
|  |  |  |  |  | MC.SaG.3 | *b* | *hz* | b | Cor1 | MH304646 | MH304690 |
|  |  |  |  |  | MC.SaG.4 | *b* | *hz* | b | Cor12 | MH304657 | MH304691 |
|  |  |  |  |  | MC.SaG.5 | b | b | b | Cor15 | MH304660 | MH304692 |
|  |  |  |  |  | MC.SaG.6 | b | b | b | Cor1 | MH304646 | MH304693 |
| Lazio | Roma | Settebagni, Parco della Marcigliana | 8 | 14 | RM.Set.1 | *b* | *hz* | i | Cor1 | MH304646 | MH304694 |
|  |  |  |  |  | RM.Set.2 | **b** | **t** | i | Cor1 | MH304646 | MH304695 |
|  |  |  |  |  | RM.Set.3 | b | b | i | Cor1 | MH304646 | MH304696 |
|  |  |  |  |  | RM.Set.4 | b | b | i | Cor1 | MH304646 | MH304697 |
|  |  |  |  |  | RM.Set.5 | b | b | i | Cor1 | MH304646 | MH304698 |
|  |  |  |  |  | RM.Set.6 | *b* | *hz* | i | Cor1 | MH304646 | MH304699 |
|  |  |  |  |  | RM.Set.7 | *b* | *hz* | i | Cor1 | MH304646 | MH304700 |
|  |  |  |  |  | RM.Set.8 | **b** | **t** | i | Cor1 | MH304646 | MH304701 |
|  |  |  |  |  | RM.Set.9 | *b* | *hz* | i | Cor1 | MH304646 | MH304702 |
|  |  |  |  |  | RM.Set.10 | *b* | *hz* | i | Cor1 | MH304646 | MH304703 |
|  |  |  |  |  | RM.Set.11 | *b* | *hz* | i | Cor1 | MH304646 | MH304704 |
|  |  |  |  |  | RM.Set.12 | b | b | i | Cor1 | MH304646 | MH304705 |
|  |  |  |  |  | RM.Set.13 | *b* | *hz* | i | Cor1 | MH304646 | MH304706 |
|  |  |  |  |  | RM.Set.14 | *b* | *hz* | i | Cor1 | MH304646 | MH304707 |
| Lazio | Roma | Gerano, Torrente Fiumicino | 9 | 4 | RM.GeG.1 | b | b | i | Cor1 | MH304646 | MH304708 |
|  |  |  |  |  | RM.GeG.2 | b | b | i | Cor7 | MH304652 | MH304709 |
|  |  |  |  |  | RM.GeG.3 | *b* | *hz* | i | Cor7 | MH304652 | MH304710 |
|  |  |  |  |  | RM.GeG.4 | b | b | i | Cor1 | MH304646 | MH304711 |
| Lazio | Roma | Gerano, Rocca Santo Stefano,Fosso dell'Orticara | 10 | 4 | RM.GeR.1 | b | b | i | Cor1 | MH304646 | MH304712 |
|  |  |  |  |  | RM.GeR.2 | *b* | *hz* | i | Cor1 | MH304646 | MH304713 |
|  |  |  |  |  | RM.GeR.3 | b | b | i | Cor1 | MH304646 | MH304714 |
|  |  |  |  |  | RM.GeR.4 | **b** | **t** | i | Cor1 | MH304646 | MH304715 |
| Lazio | Frosinone | Rocca d'Arce | 11 | 7 | FR.RoA.1 | t | - | i | Cor11 | MH304656 |  |
|  |  |  |  |  | FR.RoA.2 | *b* | *hz* | i | Cor1 | MH304646 | MH304716 |
|  |  |  |  |  | FR.RoA.3 | t | t | i | Cor11 | MH304656 | MH304717 |
|  |  |  |  |  | FR.RoA.4 | t | t | i | Cor11 | MH304656 | MH304718 |
|  |  |  |  |  | FR.RoA.5 | t | t | i | Cor11 | MH304656 | MH304719 |
|  |  |  |  |  | FR.RoA.6 | t | t | i | Cor11 | MH304656 | MH304720 |
|  |  |  |  |  | FR.RoA.7 | **b** | **t** | i | Cor1 | MH304646 | MH304721 |
| Molise | Campobasso | Guardiaregia, Torrente Vallone Grande | 12 | 1 | CB.Gua.1 | t | - | i | Cor11 | MH304656 | - |
| Campania | Caserta | Prata Sannita, Lete River | 13 | 2 | CE.PrS.1 | t | t | t | Cor10 | MH304655 | MH304722 |
|  |  |  |  |  | CE.PrS.2 | t | t | t | Cor11 | MH304656 | MH304723 |
| Puglia | Barletta | Barletta, Spinazzola | 14 | 1 | BT.Bar.1 | t | t | t | Cor19 | MH304664 | MH304724 |
| Campania | Salerno | Laurino, Calore River | 16 | 4 | SA.Lau.1 | t | t | t | Cor10 | MH304655 | MH304725 |
|  |  |  |  |  | SA.Lau.2 | t | t | t | Cor11 | MH304656 | MH304726 |
|  |  |  |  |  | SA.Lau.3 | t | t | t | Cor10 | MH304655 | MH304727 |
|  |  |  |  |  | SA.Lau.4 | t | t | t | Cor10 | MH304655 | MH304728 |
| Campania | Salerno | Morigerati, Torrente Bussento | 17 | 1 | SA.Mor.1 | t | t | t | Cor10 | MH304655 | MH304729 |
| Basilicata | Potenza | Lauria, Seluci | 18 | 3 | PZ.Lau.1 | t | t | t | Cor20 | MH304665 | MH304730 |
|  |  |  |  |  | PZ.Lau.2 | t | t | t | Cor11 | MH304656 | MH304731 |
|  |  |  |  |  | PZ.Lau.3 | t | t | t | Cor10 | MH304655 | MH304732 |
| Basilicata | Potenza | Marsicovetere, Chiuppo, Agri River | 15 | 3 | PZ.Mar.1 | t | t | t | Cor18 | MH304663 | MH304733 |
|  |  |  |  |  | PZ.Mar.2 | t | t | t | Cor10 | MH304655 | MH304734 |
|  |  |  |  |  | PZ.Mar.3 | t | t | t | Cor10 | MH304655 | MH304735 |
| Basilicata | Potenza | San Severino Lucano, Peschiera River | 19 | 6 | PZ.SSL.1 | t | t | t | Cor6 | MH304651 | MH304736 |
|  |  |  |  |  | PZ.SSL.2 | t | t | t | Cor10 | MH304655 | MH304737 |
|  |  |  |  |  | PZ.SSL.3 | t | t | t | Cor10 | MH304655 | MH304738 |
|  |  |  |  |  | PZ.SSL.4 | t | t | t | Cor11 | MH304656 | MH304739 |
|  |  |  |  |  | PZ.SSL.5 | t | t | t | Cor10 | MH304655 | MH304740 |
|  |  |  |  |  | PZ.SSL.6 | t | t | t | Cor10 | MH304655 | MH304741 |
| Calabria | Cosenza | Orsomarso | 21 | 3 | CS.Ors.1 | t | t | t | Cor10 | MH304655 | MH304742 |
|  |  |  |  |  | CS.Ors.2 | t | t | t | Cor10 | MH304655 | MH304743 |
|  |  |  |  |  | CS.Ors.3 | t | t | t | Cor10 | MH304655 | MH304744 |
| Calabria | Cosenza | Papasidero, Lao River | 20 | 1 | CS.Pap.1 | t | t | t | Cor11 | MH304656 | MH304745 |
| Calabria | Vibo Valentia | Mongiana | 22 | 3 | VV.Mon.1 | t | t | t | Cor16 | MH304661 | MH304746 |
|  |  |  |  |  | VV.Mon.2 | t | t | t | Cor14 | MH304659 | MH304747 |
|  |  |  |  |  | VV.Mon.3 | t | t | t | Cor17 | MH304662 | MH304748 |
| Sicilia | Messina | Cesarò, Nebrodi, Maulazzo | 23 | 2 | ME.Ces.1 | t | t | t | Cor9 | MH304654 | MH304749 |
|  |  |  |  |  | ME.Ces.2 | t | t | t | Cor9 | MH304654 | MH304750 |
| Sicilia | Messina | Rocella Valdemone, Torrente Licopedi | 24 | 3 | ME.RoV.1 | t | t | t | Cor8 | MH304653 | MH304751 |
|  |  |  |  |  | ME.RoV.2 | t | t | t | Cor9 | MH304654 | MH304752 |
|  |  |  |  |  | ME.RoV.3 | t | - | t | Cor8 | MH304653 | - |
| Sicilia | Siracusa | Sortino, Valle dell'Anapo | 25 | 3 | SR.Sor.1 | t | t | t | Cor13 | MH304658 | MH304753 |
|  |  |  |  |  | SR.Sor.2 | t | t | t | Cor8 | MH304653 | MH304754 |
|  |  |  |  |  | SR.Sor.3 | t | t | t | Cor8 | MH304653 | MH304755 |
| Sicilia | Ragusa | Irminio River | 26 | 1 | RG.Rag.1 | t | t | t | Cor8 | MH304653 | MH304756 |

**Table S1.** Sample details. Sampling localities, Region and Province (*Locality*); Number of the locality on the map (*map*); number of individuals per locality (*n*); Voucher code for each individuals (*voucher*); Species identification (b = *C. boltonii*; t = *C. trinacriae*; I = intermediate morphology hz= heterozygote) with mitochondrial (*coI*), nuclear (*ef1*-*α*) markers and morphological identification on field (*field*). *CoI* haplotype of GenBank sequences (*coI hp*) and Accession Numbers (*AN*).
